# Production losses associated with premature mortality in 28 European Union countries

**DOI:** 10.7189/jogh.09.020418

**Published:** 2019-12

**Authors:** Błażej Łyszczarz

**Affiliations:** Nicolaus Copernicus University in Toruń; Faculty of Health Sciences; Department of Public Health, Bydgoszcz, Poland

## Abstract

**Background:**

There has been a growing interest in the economic burden of mortality; however, a majority of evidence is concerned with particular diseases. Less is known on the overall cost of all-cause early deaths, principally in international context. Therefore, this study aims to estimate production losses of premature mortality across 28 European Union (EU-28) countries in 2015.

**Methods:**

The human capital method was applied to estimate the production losses (indirect costs) of all-cause deaths occurring at working age. The sex- and age-specific data on the number of deaths were taken from Eurostat’s database and a set of labour market measures was used to determine time of work during whole lifespan in particular countries.

**Results:**

The total production losses of all-cause premature mortality in EU-28 in 2015 were €174.6 billion, adjusted for purchasing power parity. The per capita production losses associated with early deaths were €342.39 for the whole EU-28 population on average; Lithuania experienced the highest per capita burden (€643.68), while the average costs were lowest in Greece (€188.69). These figures translated to an economic burden of 1.179% of gross domestic product in EU-28 and this share ranged from 0.679% in Luxembourg to 3.176% in Latvia. Most of the losses were due to men’s deaths and the proportion of losses associated with male mortality ranged from 64.7% in the Netherlands to 81.2% in Poland.

**Conclusions:**

Premature mortality is a considerable economic burden for European societies; however, the production losses associated with early deaths vary notably in particular countries.

There were more than 5 million deaths in the European Union (EU) in 2015 and the standardised death rates ranged from 8.6 deaths per 1000 inhabitants in France to 16.6 per 1000 in Bulgaria [1]. Around 17% of these deaths in the EU referred to people aged less than 65 [2]. These premature death cases were not only a substantial public health burden, they also resulted in explicit losses for the European economies. An evident consequence of premature mortality is a decrease of potential labour supply and this result is particularly important in the European context because of population aging. Labour shortages in the EU have already started to threaten the economic growth in some countries [3] and are expected to become more severe in the future because an increasing share of the European population ages and retires from the labour market [4]. In this context, preventing premature mortality seems to be not only a public health priority but also one of actions aimed at sustaining economic viability of the European states. Yet, our knowledge on the economic losses resulting from early deaths is limited and requires research on the identification of this burden’s magnitude, particularly in international context.

Several studies have attempted to assess the economic burden of premature mortality using the cost-of-illness approach [5-7] to quantify the production losses (indirect costs) associated with early deaths. Most of these studies are concerned with disease-specific estimates and they focus mostly on cancer [8-12] and cardiovascular diseases [13,14], two leading causes of death in developed countries. On the other hand, there are only a few studies that have analysed the costs of all-cause mortality; one of the recent papers investigated productivity impacts of all premature deaths in Australia using a microsimulation model [15], while a Columbian study assessed the costs of those mortality cases that can be considered avoidable [16]. A paper by Menzin and colleagues [17] seems to be the only research that investigates the costs of all-cause premature deaths in international context. They have developed a model for estimating the productivity losses due to premature deaths and have applied it to estimate the present value of lifetime future earnings in 29 selected developed and emerging countries. The main aim of their paper is to develop universal methods for assessing the economic burden of early deaths which can be used in the context of various countries and the authors do not compare the burden of all deaths internationally. Using a range of diversified data sources and different cost stratification (overall, by age and/or by sex) for particular countries, as well as expressing the results in national currencies, makes the findings hardly comparable across the states investigated. Moreover, the estimates of average losses per death do not show the overall economic burden of premature deaths in these countries.

Thus, to my knowledge, there is no available evidence on the production losses attributable to all-cause premature mortality that would allow for comparing these losses in a wider international context using consistent and uniform methods for a group of countries.

To fill this gap, this study aims to estimate and compare the production losses (indirect costs) associated with all-cause premature mortality in 28 EU countries (EU-28) in 2015. The knowledge of the economic burden attributable to early deaths in the European economies is important because it provides data that can be used in prioritizing public policy choices. The magnitude of the premature mortality indirect costs in particular countries shows the extent of the economic burden that can be tackled with actions aimed at improving health. Just as cost-of-illness research in particular diseases assists in prioritizing public health problems, the economic analyses of all-cause mortality provide information useful in general public decision-making, not limited to health issues solely. With this approach, the costs of early deaths and the benefits of limiting mortality through health interventions can be compared with other public actions aimed at improving social well-being by education, security, working conditions or environmental actions. This study aims to deliver internationally comparable estimates on one side of such cost-benefit relationship by providing data on potential production losses associated with early deaths.

Therefore, the contribution of this research is as follows. It is the first study that provides estimates on the indirect costs of premature deaths in a wide range of European countries. Additionally, these estimates are highly comparable across the countries because they benefit from the consistency of the data collection system in the EU allowing to use uniform methods for all states investigated. Moreover, the research offers some methodological advances as compared to the previous studies by combining contemporarily state-of-art methods and proposing some improvements in this respect.

## METHODS

The study uses cost-of-illness methodology based on retrospective data, prevalence-based top-down approach [5-7], societal perspective [18-20] and human capital method (HCM) to estimate the production losses (indirect costs) associated with premature mortality in 28 EU countries in 2015. Because the purpose of this study is to estimate the value of market production lost due to early deaths, I defined premature mortality as those cases of death that occurred at working age. However, in public health literature the concept of premature mortality is defined in various ways; depending on the purpose and scope of a research different age thresholds for the prematurity of death are used [15,21]. With the approach used here, any production lost because of deaths of those who retired was not included in the cost estimates. Also, the study only identifies the costs borne in formal economy, as no reliable data on the valuation of informal economic output (eg, housekeeping activities) exists for a wide range of countries considered here. By using the HCM, the indirect costs of premature mortality were proxied by a discounted value of production that would be produced if those who died prematurely were still alive and working until the average age of retirement [5-7]. Per worker gross domestic product calculated using Eurostat’s database [22] was used as a measure of the economies’ productivity and the estimates were adjusted for the decreasing marginal productivity of labour by applying a 0.65 correction coefficient. Generally, the use of marginal (not average) productivity is a preferred choice in indirect costs estimation [23,24] and for this reason I used this coefficient to account for the condition that – according to the law of diminishing marginal productivity – each incremental worker produces output that is lower than average. This lower productivity results from the fact that production depends on several factors and diminishing one of them (labour) affects only a respective proportion of the production output [25,26]. Therefore, the productivity lost due to premature deaths should not be considered to be as high as the average output in the economy [27]. The value of 0.65 used here reflects the proportion of output attributable to labour by applying a relationship between marginal and average labour productivity; the value of 0.65 approximates the output elasticity of labour in Cobb-Douglas production function as used in the European context [28].

Unless stated otherwise, all the input data refer to the year 2015.

In the first step of the cost estimation, the sex-specific number of deaths at each age from 0 years up to an average labour exit age in each country was identified using Eurostat’s data on mortality [2]. Because in the case of low-populated countries the number of deaths at some ages was low and unstable in time I used the average number of deaths throughout the 3-year period of 2014-2016 (For example, the number of deaths at the age of 1 year in Denmark was 8 in 2014; 2 in 2015 and 11 in 2016, and these figures exhibit large time variation in mortality cases. For this reason using a 3-year average seems to be a better choice than data from one specific year as such an average accounts for unusual variation in mortality). This allowed to reduce the impact of time variability in the numbers of deaths for those ages in which death occurs rarely. A half-cycle adjustment was applied with an assumption that all deaths occurred in the middle of the year [16].

The next step was to identify the average time a person at each age would work if had not died; for this purpose a set of following sex-specific labour market indicators for each country was used: the average age of starting first regular job (data for Denmark and Sweden were not available and for these two countries I used a sex-specific average value for the remaining 26 EU countries; data obtained from Eurostat on request); the average effective age of exit from labour market (data for the year 2016) [29]; and the employment rate for population aged 15-64 [30]. Because of the uncertainty of labour market trends, I assumed no changes in the future values of: the age of starting first job; the exit age; and the employment rates. Using these labour measures I identified the average time of work lost due to premature death separately for men and women at every working age for each of the 28 states. The following example of Portuguese females (Slovenian males) shows the calculations for women (men) with the average longest (shortest) period of economic activity throughout whole life. In 2015 women in Portugal (men in Slovenia) started their first regular job at the age of 17.73 years (20.76 years) and they are expected to work until 64.1 (60.9) years on average. Therefore, Portuguese women (Slovenian men) born in 2015 are assumed to start their job in 2032 (2035) and work 46.37 years (40.14 years) until the year 2078 (2075). Applying the employment rate of 61.1% for women (69.2% for men) to all age-specific mortality cases in the country I obtained estimates of productive time lost because of premature mortality of women (men) in Portugal (Slovenia).

The average value of production lost because of death was proxied by per worker GDP. To allow for direct comparability of the results between the countries I used this measure adjusted for purchasing power parity (PPP) [31,32] and this allowed to take into account price differences among the EU-28 countries. Future costs were discounted using a 5% rate while country-specific potential GDP per worker growth rates for each decade [29] were used to reflect economic growth. No sex- or age-specific data on per worker GDP were available; thus, the following estimates average the losses using data for whole populations and do not reflect variation in productivity of those dying prematurely.

**Figure 1:**
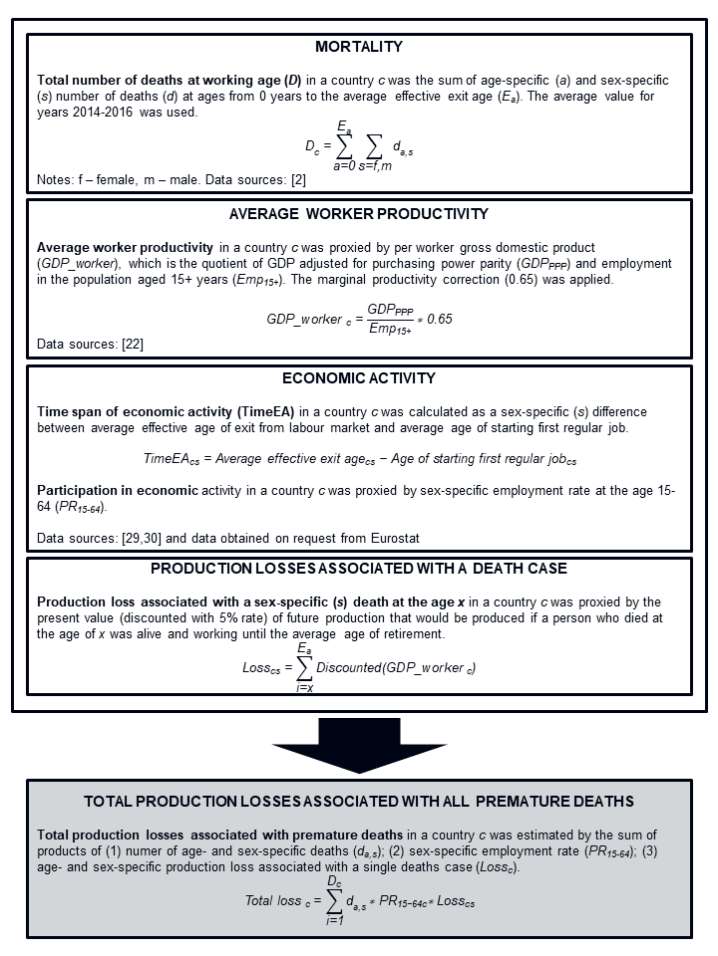
Procedure for estimation of production losses attributable to premature mortality in 28 EU countries in 2015.

**Table 1** lists the main parameters of the model for estimating production losses attributable to all-cause premature mortality in EU-28. It also shows descriptive statistics as well as the countries with minimum and maximum values for each variable used. **Figure 1** depicts the steps of the production losses estimation. The full data set used in the analysis and the example source code for one of the countries (Belgium) are available online in Appendix S1 in **Online Supplementary Document****.**

**Table 1:** Parameters of model for estimating production losses attributable to premature mortality in 28 European Union countries

Parameter (unit)	Minimum	Maximum	Average	SD
Deaths at ages from 0 to average effective exit age (number per 1000 population)*				
-Males	1.29 (Luxembourg)	5.85 (Lithuania)	2.64	1.25
-Females	0.61 (Luxembourg)	1.85 (Latvia)	1.11	0.34
Gross domestic product (million € PPP)	12 027.0 (Malta)	2 949 101.8 (Germany)	528 644.3	741 160.4
Per worker gross domestic product (€ PPP)	32 385 (Bulgaria)	171 242 (Luxembourg)	65 689	26 960
Economy's yearly productivity growth for period 2016-2070 (%)^†^	1.1 (Italy)	2.7 (Latvia)	1.6	0.4
**Average age of first regular job (years)**				
-Males	16.7 (Portugal)	21.7 (Netherlands)	19.3	1.1
-Females	17.7 (Portugal)	22.2 (Netherlands)	19.9	1.2
Average effective exit age (years)				
-Males	60.4 (Luxembourg)	65.9 (Sweden)	63.5	1.4
-Females	60.0 (Luxembourg)	65.0 (Estonia)	62.6	1.5
**Employment rate, 15-64 years (%)**				
-Males	59.3 (Greece)	79.0 (Netherlands)	70.3	5.3
-Females	42.5 (Greece)	74.0 (Sweden)	60.6	7.5

SD – standard deviation, PPP – adjusted for purchasing power parity

*The number of deaths for each particular age is an average value for years 2014-2016.

†As no predictions of economies’ growth were available for the period beyond 2070 it was assumed that growth rates in years 2070+ were the same as for the last period available (2070).

One-way deterministic sensitivity analysis was performed to assess how changes in the model’s parameters affect the results. I used a 3.5% discount rate and no discounting instead of the 5% rate in the base scenario; ±0.05 variation of 0.65 correction coefficient; gross value added instead of GDP as a measure of productivity; 0% and 2% future economic growth for all the countries; and employment rates for country-specific populations aged 15-59 years instead of 15-64 years. To account for uncertainty in future labour market trends two scenarios were used: (1) average EU values of labour measures for all the countries – labour market entry (19.34 years for males and 19.91 years for females) and labour market exit (63.5 years for males and 62.6 years for females), and (2) ages 18 and 67 used as the ages of labour market entry and exit, respectively, uniformly for all member states. Furthermore, a scenario was tested in which all the deaths occurred among minimally productive workers (Minimal productivity was obtained by dividing minimum wage by average wage in particular economies. Because there was no minimum wage legislation in six EU countries in 2015 (Denmark, Italy, Cyprus, Austria, Finland, Sweden), the weighted (population size) mean value for the remaining countries was used for the states with no minimum wage; this mean was 39.3% of the average wage). This reflects the fact that mortality is higher among lower socio-economic (SES) classes [33-36]. However, this is a scenario which shows the extreme possible lower bound of total losses and is most plausibly unrealistic; it was included because no more accurate approximation of mortality distribution by SES was available.

The other potential option to be included in sensitivity analysis was to differentiate between male and female productivity. However, I decided not to do so, because no data on per worker GDP broken down by sex was available; the only available sex-specific data was gender pay gap but it is argued that this gap reflects rather labour market discrimination than real productivity differences [37,38].

The analyses in this study were conducted using Microsoft Office Excel 2019 (Microsoft Inc, Seattle WA, USA). The study did not involve any human participants; it relied solely on publicly available data collected for other purposes. Therefore, no approval from an ethics committee was sought.

## RESULTS

The total indirect costs of all-cause premature mortality in EU-28 in 2015 were €174.6 billion adjusted for PPP (all the following costs are expressed in € PPP). More than 20 percent of the working age deaths economic burden in EU-28 was associated with premature deaths in Germany (€35.1 billion) and two other countries with the highest costs were the United Kingdom (€24.1 billion) and France (€20.9 billion). The total costs were lowest in Malta (€111.9 million) and Cyprus (€163.7 million). These differences in the total costs clearly reflect a variation in the population of the countries and it is rather the per capita cost that tells more about the economic burden of premature mortality. Per capita production losses associated with early deaths were €342.39 for the whole EU-28 population on average and the highest burden was experienced in three Baltic republics (Lithuania €643.68, Latvia €590.17, and Estonia €494.09) as well as in Ireland (€558.77) and Luxembourg (€519.74). The per capita cost in the three countries with the lowest burden of premature mortality was more than three times smaller than in Lithuania, and these were Greece (€188.69), Cyprus (€192.52) and Croatia (€208.16). In all the countries most of the costs were due to men’s deaths and the proportion of losses associated with males’ mortality ranged from 64.7% in the Netherlands to 81.2% in Poland (**Table 2**).

**Table 2:** Total and per capita production losses attributable to premature mortality in 28 European Union countries in 2015*

	Per capita costs, € PPP	Total costs, €1000 PPP (share of losses attributable to male mortality)
**Central and Eastern Europe**	**395.30**	**35 890 289 (78.6%)**
Romania	448.36	8 861 041 (78.0%)
Poland	415.98	15 797 668 (81.2%)
Hungary	330.67	3 251 422 (74.9%)
Bulgaria	362.24	2 600 145 (73.3%)
Slovakia	359.80	1 953 180 (76.2%)
Czech Republic	324.94	3 426 833 (77.0%)
**Northern Europe**	**392.30**	**38 049 512 (67.8%)**
Lithuania	643.68	1 858 867 (78.6%)
Latvia	590.17	1 162 491 (71.2%)
Ireland	558.77	2 642 562 (68.6%)
Estonia	494.09	650 625 (74.4%)
Denmark	401.28	2 280 684 (66.7%)
United Kingdom	369.20	24 141 508 (66.5%)
Sweden	347.41	3 428 839 (66.6%)
Finland	343.17	1 883 935 (71.3%)
**Southern Europe**	**231.41**	**31 452 749 (70.5%)**
Portugal	273.07	2 822 877 (71.7%)
Malta	248.23	111 864 (75.5%)
Spain	240.70	11 180 048 (68.1%)
Italy	227.68	13 820 880 (71.0%)
Slovenia	214.87	443 692 (74.0%)
Croatia	208.16	872 390 (76.1%)
Cyprus	192.52	163 659 (69.2%)
Greece	188.69	2 037 340 (75.9%)
**Western Europe**	**372.51**	**69 177 789 (68.5%)**
Luxembourg	519.74	299 513 (72.1%)
Germany	429.18	35 058 290 (68.8%)
Netherlands	365.01	6 201 856 (64.7%)
Austria	363.35	3 160 712 (71.4%)
Belgium	318.49	3 590 702 (66.6%)
France	312.74	20 866 717 (68.8%)
**European Union**	**342.39**	**174 570 339 (70.8%)**

PPP – adjusted for purchasing power parity

*The mean value for per capita costs and share of losses attributable to male mortality for each of the four regions are population-weighted averages.

Considering the sub-regions of Europe, the per capita cost was notably lower in the southern countries (€231.41) comparing to the other three regions where the losses were of similar magnitude (€395.30 in the central and eastern; €392.30 in the northern; and €372.51 in the western states). The share of total costs attributable to male mortality was highest in Central and Eastern Europe (CEE) (78.6%) while in the other three regions it ranged between 67.8% and 70.5% (**Table 2**).

**Figure 2:**
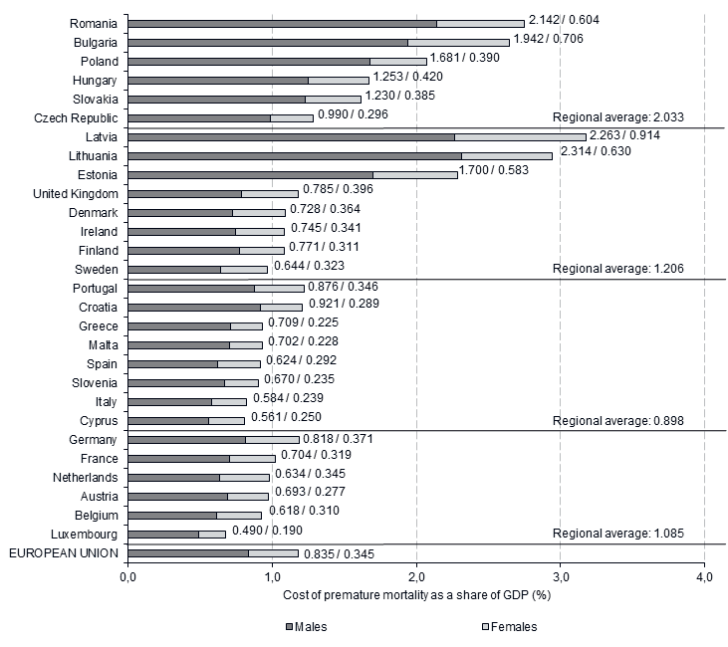
Production losses attributable to premature mortality in 28 EU countries as a share of GDP (2015).

**Figure 2** shows sex-specific indirect costs of premature mortality in EU-28 as a percentage of GDP allowing to assess burden in relation to the economic potential of particular countries.

The share of GDP lost due to premature mortality in the whole EU economy was 1.179%. Six countries lost more than 2% of GDP and the burden was highest in three Baltic states (Latvia 3.176% of GDP; Lithuania 2.944%, and Estonia 2.283%) as well as in Romania (2.746%) and Bulgaria (2.648%), two relatively less economically developed countries which, at the same time, have the highest mortality rates across the EU. On the other hand, in the three countries with the lowest losses, the indirect costs were lower than 0.9% of GDP and Luxembourg was a state with the burden of only 0.679% of GDP, followed by Cyprus (0.811%) and Italy (0.823%) (**Figure 2**).

The regional analysis shows that the relative burden of early deaths was highest in the CEE states (2.033% of regional GDP), followed by the northern (1.206% of GDP), western (1.085% of GDP) and southern countries (0.898% of GDP). However, there was a notable heterogeneity of GDP-based measure among the northern states; the three Baltic countries experienced much higher losses than the other countries of the region. A large variation was also observed among the CEE countries while the results were more homogenous for the groups of the southern and western states (**Figure 2**).

**Figure 3:**
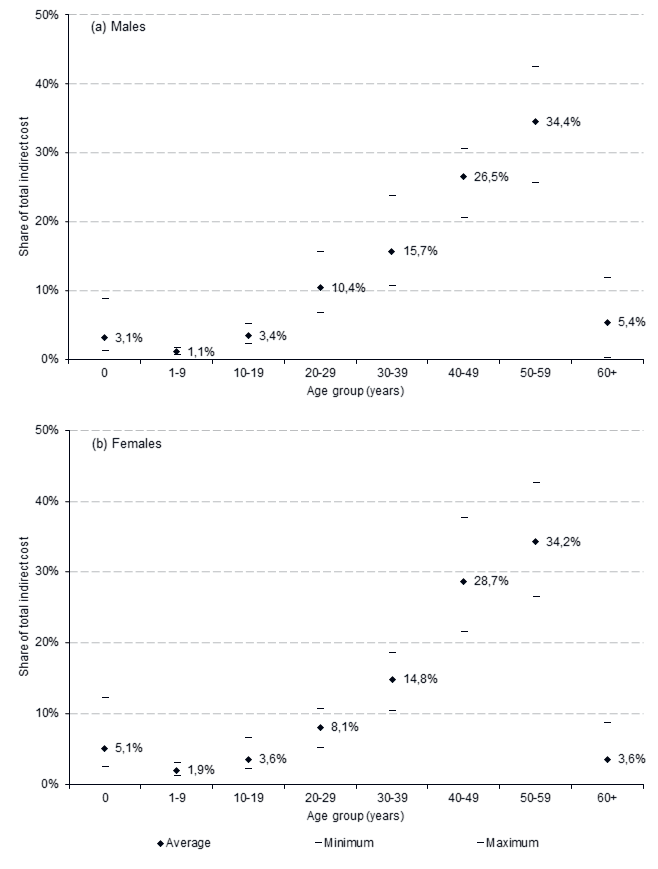
Age distribution of (a) male and (b) female production losses attributable to premature mortality in 28 European Union countries (2015). Notes: Averages show unweighted mean values for all EU-28 countries.

The (EU-average) age distribution of premature mortality losses shows that more than 60% of the total costs were associated with deaths of those at their 40s and 50s. The magnitude of infant deaths (0 years of age) was higher in women than in men (5.1% vs 3.1% of total costs on average), while the burden of deaths in the population aged 60+ was relatively greater for men than for women (5.4% vs 3.6%) (**Figure 3**). The description of age distribution for particular countries is beyond the scope of this paper and the interested reader is referred to Appendix S2 of **Online Supplementary Document** where detailed results are provided.

The results of one-way deterministic sensitivity analysis show that my estimates are prone to changes in the model’s parameters, with the greatest variation in the costs estimated resulting from changes in the discount rate (**Table 3**). Using the 3.5% discount rate, instead of 5% (base scenario), elevated the costs by 15.5% on average, with a variation between countries ranging from 13.5% (Croatia) to 19.9% (Malta). With no discounting, the indirect costs were 84.7% higher on average and they more than doubled for some countries (Ireland, Malta and Romania). Varying the value of coefficient adjusting for decreasing marginal productivity by ±0.05 changed the estimates by ±7.7%. Using gross value added as a measure of economy productivity decreased the estimates by 11.6% on average, with the minimum change observed for Ireland (-7.4%) and the maximum change for Croatia (-16.0%). Assuming that all the deaths were among those minimally productive resulted in losses lower by 59.8% on average; however, this result shows an extreme value which is implausible and it rather gives an extreme thinkable estimate. The assumption of 0% (2%) future economic growth varied the results by -11.5% (3.3%) on average, while using employment rates for those aged 15-59 years elevated the results from 1.2% (Sweden) to 8.0% (Slovenia). The use of alternative input data on labour market characteristics had an important effect on the results; with the entry age of 18 and exit age of 67 for both sexes the results changed by 39.0% on average with the highest (lowest) change of 81.3% (13.6%) in Slovenia (Sweden). On the other hand, when the average EU values for these measures were used for all the countries, the changes were not that notable (**Table 3**). A detailed, country-specific sensitivity analysis is available online in Appendix S3 of **Online Supplementary Document**.

**Table 3:** Summary of sensitivity analysis for estimates of production losses attributable to premature mortality in 28 European Union countries

BS – base scenario (results shown in Table 2)				
	Average* change from BS	**Minimum change from BS**		**Maximum change from BS**
**Discount rate (BS = 5%):**				
3.5%	15.5%	13.5% (Croatia)		19.9% (Malta)
0%	84.7%	71.3% (Italy)		120.7% (Malta)
**Coefficient to adjust for marginal labour productivity (BS = 0.65):**				
0.6	-7.7%		-7.7% (all countries)	
0.7	7.7%		7.7% (all countries)	
**Productivity measure (BS = gross domestic product):**				
Gross value added	-11.6%	-7.4% (Ireland)		-16.0% (Croatia)
**Minimum productivity adjustment^†^ (BS = average productivity)**	-59.8%	-47.6% (Slovenia)		-65.7% (Spain)
**Future economic growth (BS = country-specific):**				
0% for all the countries	-11.5%	-1.8% (Italy)		-28.3% (Latvia)
2% for all the countries	3.3%	0.2% (Hungary)		-16.7% (Latvia)
**Employment rate (BS = 15-64 years):**				
15-59 years	4.4%	1.2% (Sweden)		8.0% (Slovenia)
**Labour market entry and retirement age (BS = country-specific):**				
Sex-specific, average EU values	0.9%	-0.5% (Poland)		28.3% (Luxembourg)
Both sexes: 18 years and 67 years	39.0%	13.6% (Sweden)		81.3% (Slovenia)

*Unweighted average.

†Minimum productivity adjustment assumes that all deaths occurred among those being minimally productive and this was proxied by the share of minimum wage in average wage in each EU economy.

All the tabulated and depicted results are available online in a spreadsheet of Appendix S4 of **Online Supplementary Document****.**

## DISCUSSION

This study estimated production losses (indirect costs) attributable to all-cause premature mortality in 28 EU countries for the year 2015. For this purpose I used a prevalence-based top-down approach, societal perspective and the HCM, and the estimates were based on uniform methods and data for all the countries investigated. Therefore, this is the first study that assessed the indirect costs of early deaths for a range of European states which allows for a direct comparison of the economic burden between the countries.

The results show a large divergence in the costs of premature mortality between the countries. The per capita costs (expressed in PPP €) were almost 3.5 times higher in the country with the greatest burden (Lithuania, €643.68) compared to the country with the lowest costs (Greece, €188.69). There was also a great variation in the economic burden of early deaths when the costs were expressed in relation to economic development; the production losses in Latvia accounted for 3.176% of GDP, while in Luxembourg this share was only 0.679% of GDP. The variation in the values of both these measures shows that the European economies were affected by premature deaths to a very different extent. Interestingly, the difference in the indirect costs was much deeper than mortality disparity; Bulgaria, a country experiencing the highest health burden of deaths in Europe had a standardised mortality rate not even twice as high as France, a country with the lowest rate (16.6 vs 8.6 deaths per 1000 population). This shows that not only mortality rates were important for the economic burden of early deaths, but also market labour and general economic conditions mattered. For example, Latvia and Lithuania not only had enormously high death rates; both these countries have much above-the-average prospects of their future economic growth, a high employment rate among women and a relatively large difference between exit age and age of starting first job. All these factors contribute to a high burden of premature deaths and underpin the mortality effect. On the other hand, the comparatively low costs observed for Greece, Cyprus or Luxembourg do not result from low mortality therein but from unfavourable economic and labour market trends, namely slow predicted economic growth in the future, low employment rates and/or low exit age. Altogether, the results show that the economic burden of premature deaths not only depends on mortality patterns; the economic conditions of particular countries are also important for the production losses experienced.

A notable result is a clear geographical pattern of economic losses; most of the countries experiencing high indirect costs were those from Central and Eastern Europe and the three Baltic states. This result should not be surprising as these states are also among the ones with the highest mortality rates. It is also predicted that their economies will grow more quickly than the Western European ones and this convergence process leads to comparatively high costs associated with future losses of current deaths.

The sex difference in the indirect costs of all-cause premature mortality reflects higher mortality rates among men across all the countries. As a result, the losses attributable to males account for 64.7%-81.2% of the total costs depending on the country. Generally, a higher share of losses attributable to male mortality was observed in the CEE countries as well as the three Baltic States. The sex disparity in costs identified here is clearly deeper than the mortality difference between men and women and can be explained by higher and longer activity rates among men in the labour markets. Considering the age distribution of production losses, the burden is relatively high for women up to their 20s, while for men the costs for those over 60 are relatively more meaningful, and this results from men’s later exit from labour market.

The sensitivity analysis conducted shows that my results are subject to meaningful changes with variation in the parameters. Particularly, changing the discount rate affects the findings notably; with no discounting, the losses estimated are 84.6% higher on average than in the base scenario. This result should not be surprising though, as the time perspective of the analysis extends several decades towards the future (up to the year 2080). Moreover, assuming that all the deaths occurred among minimally productive workers leads to ~ 60% lower estimates on average; however, it must be stressed again that this extreme result is highly implausible. The source of other important estimates’ variation was the use of an early labour entry age (18 years) and late labour exit age (67 years); the result of this sensitivity scenario was 39% higher on average than the base case and it shows by how much the indirect costs of mortality may increase with a growing time-span of economic activity in Europe.

For other modifications in the parameter values, the results are more stable; for example, when applying a 3.5% discount rate the results differ from the base scenario by 13.5%-19.9% and the changes are even lower for an alternative measure of productivity and a variation in 0.65 correction coefficient.

The present estimates could not be directly compared with the findings from other studies as, to my knowledge, there have not been any analogous estimates published until now. The study by Menzin et al. [17] only provides a value of lifetime future earnings for a single death case and does not generalize this result to the whole economy’s level. Hence, despite the fact that their study and my estimates are concerned with the same issue, the way the results are communicated differs in a way that precludes direct comparison. Another study estimates the cost of premature mortality in Australia in 2003 for $13.8 billion (using 2015 Australian dollars) [15] and this translates to 1.179% of GDP. However, this result cannot be compared to my estimates because the Australian study does not adjust the estimates for decreasing labour productivity (0.65 coefficient). With this correction applied, the production losses estimated for Australia would account for 0.766% of GDP, a burden of similar magnitude to the ones obtained for the countries with the lowest burden in EU (see **Figure 2**). The results of the Colombian study provide estimates on the costs of avoidable deaths which ranged from US $3.69 billion to US $6.89 billion, depending on the scenario [16], and this translates to 0.663% and 1.238% of GDP with a 0.65 correction applied. It needs to be emphasised though, that the Colombian findings refer to avoidable deaths while the present study is concerned with premature mortality and these two categories differ.

This study used the best methodological practices from the previous studies and proposed some methodological advances. First, it used figures on death cases for each single year of life, instead of using death cases grouped in 5-year intervals, and this allowed for more precise costs estimates. Second, this study accounted for varying future growth rates of economies across time and particular countries, and with this approach each state’s specificity was accounted for. Third, instead of using legal retirement age, the study used the real data on the effective age of exit from labour market; also differences in the age of staring first job between the countries were considered, and with the use of real labour market measures the estimates are more credible. Additionally, by adjusting the costs with PPP and expressing them in relation to GDP, the results are comparable across countries; on the other hand, the costs estimates for each country can be easily transformed to local currencies, that being more useful in a national context. Moreover, the study used a single data source (Eurostat database) and benefited from the uniformity of data collection; with this approach my estimates are highly comparable across the countries. Finally, by applying correction for decreasing marginal labour productivity (0.65), the results accounted for the fact that economic output increments diminish with each additional worker.

### Limitations of the study

The estimation of losses attributable to premature mortality was possible only with several assumptions which result in certain limitations. First, by using the average productivity measure (per worker GDP), the indirect costs estimates are potentially biased upwards because mortality rates are higher in lower socioeconomic groups that are relatively less productive [33-36]. For this reason, the mean economic output of those who died prematurely may be lower than the average per worker GDP in the economy. However, the scenario of minimum productivity adjustment in sensitivity analysis was applied to address this limitation. Moreover, the study used other average values in input data. This could have had a potentially meaningful effect for my results; the absence of sex- and age-specific values of per worker GDP could have further biased the results. Nevertheless, this shortcoming resulting from the use of average measures is not unique for the present study, as only research utilizing data on individual characteristics (like the microsimulation Australian model [15]) are able to control for socioeconomic status in premature mortality analyses. Using aggregated data for several countries makes such analysis unmanageable.

Second, the human capital approach used here is criticised [5,23,39] because it approximates potential or maximum production losses associated with premature mortality. This criticism is based on the assumption that economic loss is only limited to the friction period needed to replace a worker who died by another employee [40]. However, the friction cost method is not free from its own methodological challenges (for the review see [41]) and the HCM is still the most frequently used and accepted approach to evaluate indirect costs [42]. Third, this analysis only accounted for the losses experienced in formal economy as I was not able to estimate the indirect costs of undone housekeeping and caregiving activities or unregistered production lost because of premature deaths. Regrettably, with a wide range of countries included, this non-market production was not measurable in a comparable way. However, with growing recognition of informal activities’ economic contribution [43] and increasing data accessibility [44], this cost component will possibly be included in future international studies on indirect costs. Fourth, because a number of indicators used was population-based, the study is at risk of an ecological fallacy. This issue is not addressed in the study design of cost-of-illness analysis used here; however, some researchers point to potential bias resulting from data aggregation [45,46]. Fifth, the sensitivity analysis exhibits large variation in the estimates, particularly for the minimum productivity adjustment and different discount rates used. The choice of discount rate itself is another problem; some researchers argue that discounting should not be used in premature mortality cost analyses [47]. Finally, as this analysis spans to several decades towards the future (up to 2080), the assumptions made about future economic conditions (GDP growth, labour market trends) are fairly uncertain. To account for this uncertainty, extensive sensitivity analysis with various scenarios (including no discounting and different values of labour market measures) was conducted.

To conclude on the study limitations, the shortcomings discussed imply that caution is needed when interpreting the findings of this study. Still, these limitations are not unique for this research and they also characterize previous studies.

## CONCLUSIONS

In conclusion, this study estimated the production losses (indirect costs) associated with premature mortality in 28 European Union countries in 2015. This burden ranged from €188.69 to €643.68 (adjusted for PPP) per capita costs and these values translated to the economic losses of 0.679%-3.176% of GDP depending on a country. Clearly, premature mortality cannot be eliminated completely and these estimates do not show the gains that are possible to achieve even with the best health care provided; they rather approximate potential or maximum losses. Still, these values give a picture of economic burden experienced by European societies and provide data useful in prioritising public policy choices. The economic burden of early deaths can be compared with losses resulting from other social or environmental problems, eg, unemployment, poverty, low educational attainment or pollution. Additionally, with the estimated data, spending on the health care activities that reduce mortality can be compared with the production losses avoided because of investments in health. Such calculation seems to be particularly important in the present demographic situation in Europe where health gains should be considered not only a public health issue but also economic benefit for the aging societies.

## Additional material

Online Supplementary Document
